# Evaluating the Performance of De Novo Assembly Methods for Venom-Gland Transcriptomics

**DOI:** 10.3390/toxins10060249

**Published:** 2018-06-19

**Authors:** Matthew L. Holding, Mark J. Margres, Andrew J. Mason, Christopher L. Parkinson, Darin R. Rokyta

**Affiliations:** 1 Department of Biological Sciences, Clemson University, Clemson, SC 29634, USA; matthewholding28@gmail.com (M.L.H.); ajmason@g.clemson.edu (A.J.M.); viper@clemson.edu (C.L.P.); 2 Department of Biological Science, Florida State University, Tallahassee, FL 32306, USA; 3 School of Biological Sciences, Washington State University, Pullman, WA 99164, USA; mark.margres@wsu.edu; 4 Department of Forestry, and Environmental Conservation, Clemson University, Clemson, SC 29634, USA

**Keywords:** venom-gland transcriptome, transcriptome assembly, snake, scorpion

## Abstract

Venom-gland transcriptomics is a key tool in the study of the evolution, ecology, function, and pharmacology of animal venoms. In particular, gene-expression variation and coding sequences gained through transcriptomics provide key information for explaining functional venom variation over both ecological and evolutionary timescales. The accuracy and usefulness of inferences made through transcriptomics, however, is limited by the accuracy of the transcriptome assembly, which is a bioinformatic problem with several possible solutions. Several methods have been employed to assemble venom-gland transcriptomes, with the Trinity assembler being the most commonly applied among them. Although previous evidence of variation in performance among assembly software exists, particularly regarding recovery of difficult-to-assemble multigene families such as snake venom metalloproteinases, much work to date still employs a single assembly method. We evaluated the performance of several commonly used de novo assembly methods for the recovery of both nontoxin transcripts and complete, high-quality venom-gene transcripts across eleven snake and four scorpion transcriptomes. We varied *k*-mer sizes used by some assemblers to evaluate the impact of *k*-mer length on transcript recovery. We showed that the recovery of nontoxin transcripts and toxin transcripts is best accomplished through different assembly software, with SDT at smaller *k*-mer lengths and Trinity being best for nontoxin recovery and a combination of SeqMan NGen and a seed-and-extend approach implemented in Extender as the best means of recovering a complete set of toxin transcripts. In particular, Extender was the only means tested capable of assembling multiple isoforms of the diverse snake venom metalloproteinase family, while traditional approaches such as Trinity recovered at most one metalloproteinase transcript. Our work demonstrated that traditional metrics of assembly performance are not predictive of performance in the recovery of complete and high quality toxin genes. Instead, effective venom-gland transcriptomic studies should combine and quality-filter the results of several assemblers with varying algorithmic strategies.

## 1. Introduction

The traits mediating interactions between species have special ecological and evolutionary significance, making them the foci of studies of adaptation, innovation, and the mode and tempo of diversification [1,2,3]. Although uncovering the genetic basis of variability in these traits is often difficult, animal venoms, proteinaceous secretions from specialized glands that are directly injected into prey or enemies, present a genetically tractable model to map the progression from genotype to phenotype [4,5]. In venoms, expressed genes yield a mixture of tens to hundreds of proteins and peptides as the terminal phenotype [6,7]. Venom compositional differences in many venomous taxa are closely linked to variable gene expression [5,8], which can be quantified by RNA-seq-based transcriptomics. Transcriptomes also provide sequence data valuable for evolutionary rate assessments, gene tree construction, genome annotation, and improving the quality and completeness of proteomic characterizations of the venom itself [9,10]. However, the power of transcriptomics to provide these insights is impacted by the completeness and quality of the transcriptome assembly.

Assembling a complete transcriptome from tens to hundreds of millions of short read sequences is a non-trivial task, although a number of bioinformatic tools have been developed for this purpose. Although a reference-based framework can be used for transcriptome assembly, the lack of high-quality genomic resources for many venomous species has led most studies to rely on de novo assembly approaches. The majority of these programs are de Bruijn graph-based assemblers [11,12,13,14,15,16], which create a network of *k*-mer nodes connected by edges representing *k*-mer similarity. Contigs are recovered by traversing these edges to identify transcripts and their various isoforms. The usage of *de Bruijn* graphs for assembly allows for a dramatic increase in computational efficiency, but can also result in the recovery of misleading chimeric transcripts as bioinformatic artifacts of assembly [17,18]. Thus, resulting assemblies must be carefully checked to filter and avoid the accidental inclusion of the false sequences in the final assembly product. Of the assemblers used in venom gland transcriptomics, the *de Bruijn* based Trinity has been the most widely employed [19,20,21,22,23,24,25,26,27,28,29,30,31,32], although the integration of other assemblers is becoming more common [33,34,35,36]. Empirical studies of transcriptome assembler performance, however, have demonstrated that the use of a single assembly method rarely produces full transcriptome recovery and that taxon and tissue-specific variation in assembler performance may exist [37,38,39]. Assembly of venom-gland transcriptomes is further complicated by wide disparity in transcript expression and varying degrees of paralogy and toxin divergence that can confound transcript recovery. Trinity, in particular, is known to collapse toxin isoforms into a single transcript in the face of point mutations and minimal divergence [40], as well as to return generally shorter contigs than some other assemblers [39,41]. The main concern is that incomplete or biased recovery of transcripts at this stage will have downstream ramifications for estimating transcript abundance, diversity, and evolution. These potential complications, as well as the growing use of RNA-seq in studies of animal venoms, underscores the necessity for a critical evaluation of assembler performance for venom gland transcriptomics.

We generated de novo assemblies of RNA-seq reads using several different assembly methods to evaluate the strengths and weaknesses of each approach for the assembly of venom-gland transcriptomes across seven species of pitviper (12 total transcriptomes) and two species of scorpion (four total transcriptomes; Table 1). First, we assessed differences among assembly approaches in the recovery of nontoxin transcripts from venom glands, which can be important to studies of toxin evolution by establishing baselines for studies of diversity and evolutionary rates and for building phylogenies via transcriptome mining (e.g., [42]). Next, we evaluated each assembly using criteria for both total numbers and toxin family specific numbers of high quality (non-chimeric) toxin genes assembled. Finally, we assessed toxin-gene assembly completeness in a snake, *Crotalus adamanteus*, by annotating the contigs recovered by each assembly method using a previously published consensus set of toxin-encoding *C. adamanteus* sequences produced by Rokyta et al. [43]. We highlight the high variability in the transcripts recovered by different assembly methods and provide practical recommendations for the recovery of complete, high-quality venom-gland transcriptomes for studies of toxinology and toxin gene evolution.

## 2. Results

### 2.1. Overall Assembly Quality and Recovery of Nontoxins

**Figure 1:**
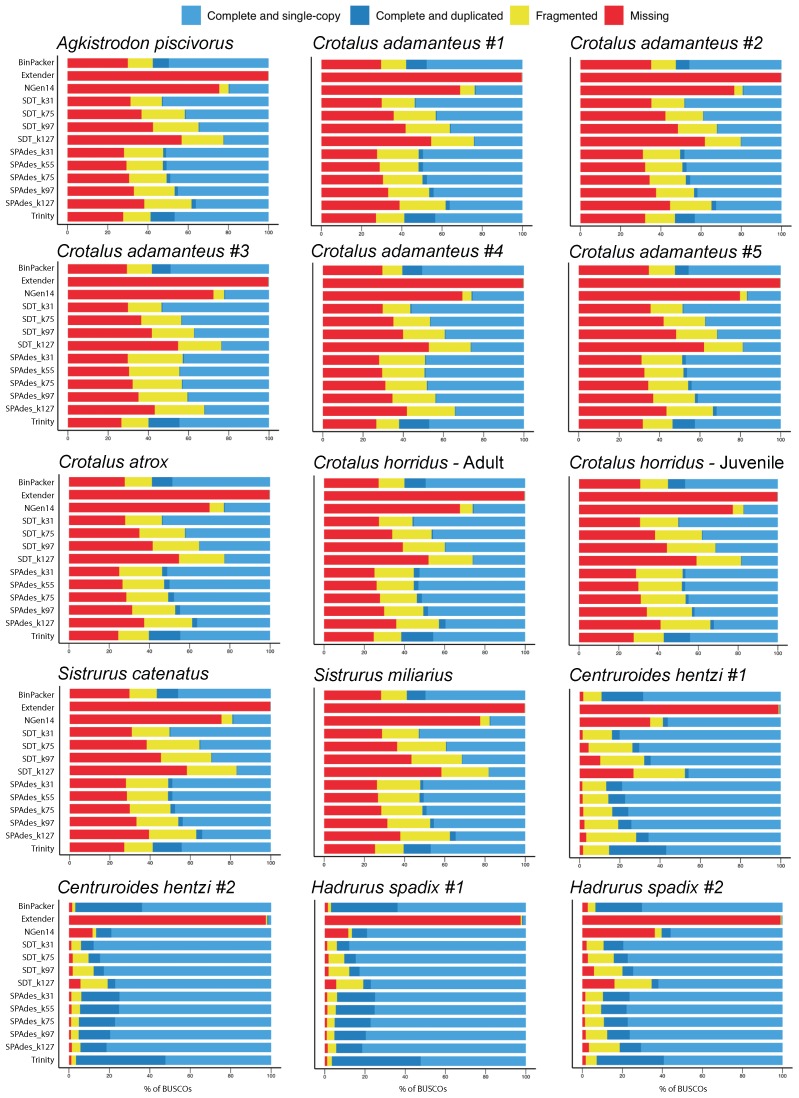
BUSCO completeness analyses of venom gland transcriptome assemblies of six snake species and two scorpion species showed that short *k*-mer approaches performed best for single-copy nontoxins. Snake contigs were matched against 3950 orthologous loci defined within Tetrapoda, while the scorpion contigs were searched against 1066 orthologous loci defined within Arthropoda.

The ability of assembly methods to recover complete sets of nontoxin transcripts, as measured by BUSCO [44], varied considerably, but clear and consistent trends in relative performance across individual transcriptomes emerged (Figure 1). SOAPdenovo-Trans [12] assemblies constructed with a *k*-mer size of 31 (SDT_k31) consistently yielded the highest number of complete and single copy nontoxin transcripts. This result held true for all snake and scorpion individuals, where snake transcriptomes assembled with SDT_k31 yielded an average of 2033 (range: 1797–2238) complete and single copy nontoxin loci out of 3950 reference loci, and scorpion transcriptomes yielded 823 (range: 652–936) out of 1066 reference loci.

The ranking of the other assembly methods differed depending on whether snakes or scorpions were being considered, but some general patterns emerged. First, increasing *k*-mer size tended to lead to fewer orthologous loci recovered by BUSCO (Figure 1). Second, BinPacker [45] was the second best method for the recovery of nontoxins in snakes and was the seventh best in scorpions, indicating that its performance will be something between middling and good. Third, Trinity [46] and NGen14 (DNAStar, Inc., Madison, WI, USA) performed comparatively poorly in both snakes and scorpions. In particular, NGen14 recovered less than half of the snake loci recovered by SDT_k31 and less than 3/4 of the scorpion loci. Extender [33] was largely ineffective at recovering nontoxin loci, recovering between two and eight complete, single-copy loci in snakes and between one and 15 in scorpions.

**Figure 2:**
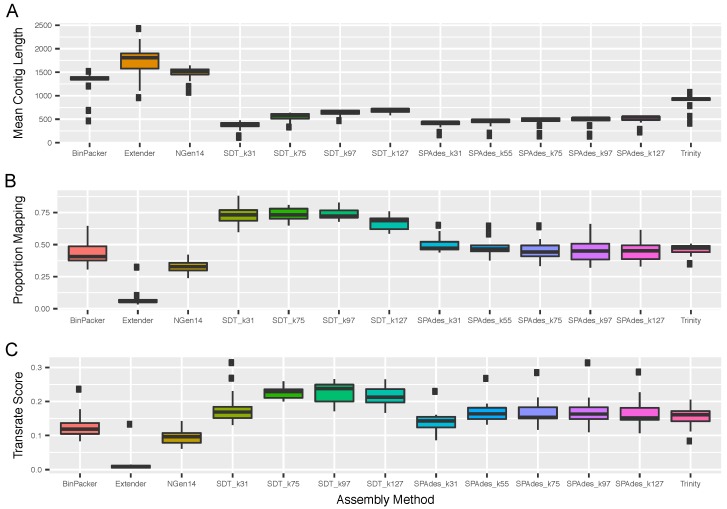
Metrics for assembled transcriptomes from Transrate showed that SDT with moderate *k*-mer sizes performed best overall. Plots show (**A**) average length of the contigs produced by each assembly method, (**B**) proportion of total reads that mapped to the the assembled transcriptome, using default Transrate mapping parameters, and (**C**) the conglomerate “Transrate Score” provided by Transrate as a metric of assembly quality. Boxplots show median and interquartile range across all snake and scorpion specimens analyzed herein.

The assembly quality statistics provided by Transrate [47] yielded similar overall rankings of performance to the BUSCO [44] completeness measures (Figure 2). In particular, SDT and SPAdes [15] produced assemblies with the highest Transrate scores and proportions of reads mapping to the assembly, with SDT_k97 producing the highest average Transrate scores. However, Transrate scores tended to increase with increasing *k*-mer size, whereas smaller *k*-mer sizes led to the identification of more loci by BUSCO. If these additional loci recovered tend to be of lower quality, researchers should consider whether intermediate or large *k*-mer sizes provide higher quality data at the cost of a few loci. As with BUSCO, Trinity and BinPacker performed moderately well as ranked by Transrate scores, whereas Extender and NGen14 performed poorly. However, Extender and NGen14 produced the highest mean contig lengths, which belies their differential performance in the targeted assembly of long toxin loci (see below).

### 2.2. Snake Toxin Assembly Quality

**Figure 3:**
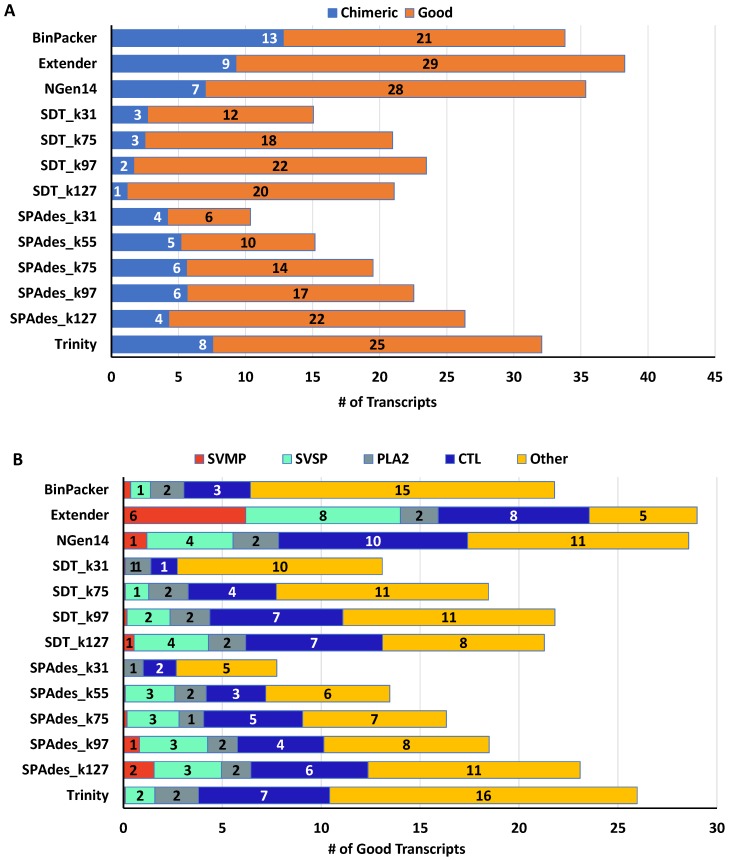
Average toxin transcript statistics among all individual snakes for each of 13 assembly approaches showed that Extender and NGen14 outperformed other methods for toxin-transcript assembly. Bars represent (**A**) transcripts classified as chimeric versus good and (**B**) the proportion of good transcripts from each of several venom gene families. The *k*-mer size for SOAPdenovo-Trans (SDT) and rnaSPAdes (SPAdes) analyses is indicated by “_k##” in the method name. Abbreviations: phospholipase A${}_{2}$ (PLA${}_{2}$), snake venom metalloproteinase (SVMP), snake venom serine protease (SVSP), C-type lectin (CTL).

Assemblers varied considerably in the recovery of quality toxin transcripts in snakes, with the toxin gene family in question and the *k*-mer sized used in SDT and SPAdes assemblies emerging as clear drivers of trends in relative performance (Table 2). The assemblers that yielded the highest mean numbers of transcripts passing our quality filters were Extender (29 transcripts), NGen14 (28 transcripts), and Trinity (25 transcripts) with a steep decline in output for the remaining approaches (Figure 3A). BinPacker produced a comparable number of total transcripts to the previously mentioned approaches, but over 1/3 of these failed to pass quality filtering.

A clear trend emerged regarding the recovery of snake venom metalloproteinase (SVMP), snake venom serine proteinase (SVSP), and C-type lectin (CTL) transcripts, which are relatively long transcripts (∼1400–1800 bp, ∼700–800 bp, and ∼400–500 bp, respectively) and exist as multigene families of >3 loci in these snakes. For these toxin families, the seed and extend approach employed through Extender greatly outpaced the other assemblers (Figure 3B). Extender recovered ∼6 SVMP paralogs per snake, whereas other approaches assembled at most one member of this important toxin family. A similar but less extreme trend existed for SVSPs, where Extender nearly doubled the recovered SVSP transcripts compared to the next-best approach in NGen14. Finally, the Extender and NGen14 were comparably effective in the recovery of CTL loci, with Trinity also performing well. The SDT, SPAdes, and BinPacker assemblers performed comparatively poorly in assembling CTL loci, although not as poorly as for SVMPs and SVSPs. Taken together, these results point toward toxin genomic complexity, particularly in terms of gene family diversity, as a key factor in determining the optimal assembly strategy for recovering high quality transcripts via de novo assemblies.

The rank performance of each assembler shifted when considering snake toxins that exist as smaller gene families or as a single locus and tend to show lower relative expression in the transcriptome compared to SVMPs and SVSPs. These toxins genes include three-finger toxins (3FTx), bradykinin-potentiating and C-type natriuretic peptides (BPP), cysteine-rich secretory proteins (CRISP), hyaluronidases (HYAL), Kunitz-type protease inhibitors (KUN), L-amino acid oxidases (LAAO), myotoxins (MYO), nerve growth factors (NGF), nucleotidases (NUC), phosphodiesterases (PDE), phospholipases A${}_{2}$ (PLA${}_{2}$), phospholipases B (PLB), vascular endothelial growth factors (VEGF), and Vespryn. Of this group, Trinity recovered the highest number of good transcripts, with similarly high performance from NGen14 and BinPacker, followed by Extender, SDT_k127 and SPADes_k127 (Figure 3B).

**Figure 4:**
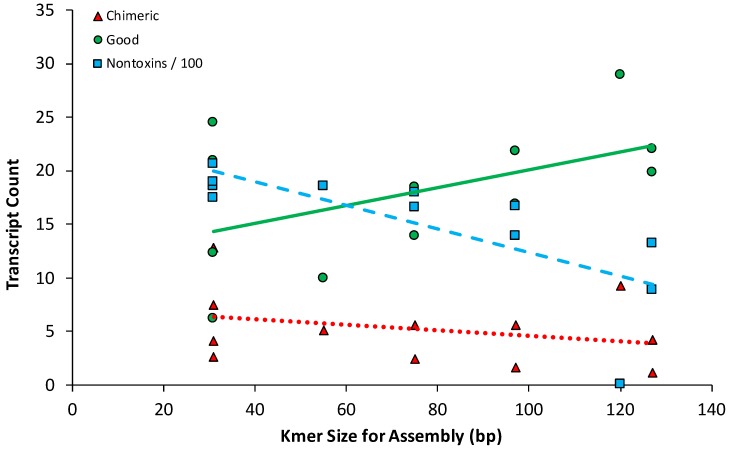
The relationship between *k*-mer size and the mean number of assembled chimeric toxins (triangles), good toxins (circles), and nontoxins (squares) across eleven snake venom transcriptomes showed the conflict between assembling nontoxins and toxins. Nontoxin values are based on the number of “complete and single copy” loci detected by BUSCO analysis divided by 100 to place them on a comparable scale with the toxin loci. The SeqMan NGen results were excluded from this analysis because it lacks a defined *k*-mer value analogous to the other methods.

Notable trends emerged regarding transcriptome quality and the *k*-mer size used by each assembly method. SOAPdenovo-trans and SPAdes analyses at lower *k*-mer sizes showed considerable performance drop-offs in the production of quality toxin genes compared to other assemblers, while tending to recover more nontoxins based on BUSCO analyses (Figure 1). Using the *k*-mer size of all assembly methods, excepting the *k*-mer-free BinPacker, we conducted simple linear regressions to further explore how *k*-mer size impacts the recovery of both toxins and nontoxins (Figure 4). There was no evidence for a relationship between *k*-mer sizes and the number of chimeras produced (*p* = 0.34), while the number of good toxin transcripts recovered tended to increase with increasing *k*-mer size, but the relationship is marginally non-significant (*p* = 0.09). Finally, smaller *k*-mer size was associated with the recovery of significantly higher numbers of nontoxin loci (*p* = 0.005).

### 2.3. Assembly Completeness for the Crotalus adamanteus Transcriptome

**Figure 5:**
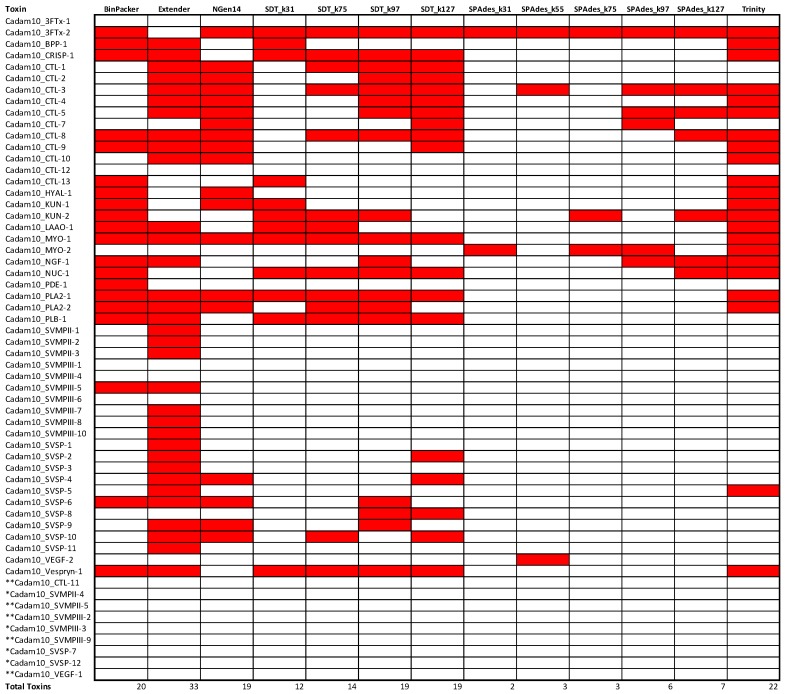
Performance comparison of each assembly method in the recovery of known transcripts for the *Crotalus adamanteus* venom gland transcriptome when assembling the transcriptome of Cadam-KW1942 using 13 different assembly methods. * indicates a known *C. adamanteus* transcript that was not detected in either Cadam-KW1942 or Cadam-KW2161, while ** indicates a known *C. adamanteus* transcript that was not detected among any of the five transcriptomes for this species analyzed in the current study.

**Figure 6:**
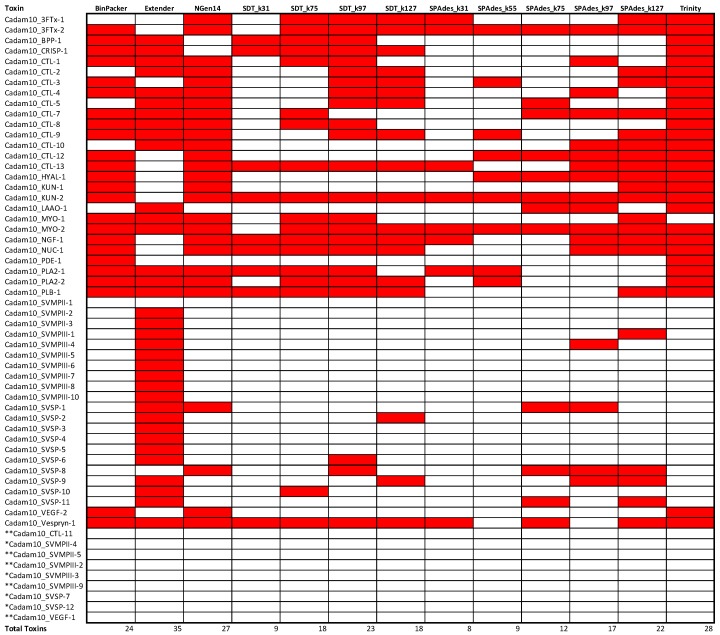
Performance comparison of each assembly method in the recovery of known transcripts for the *Crotalus adamanteus* venom gland transcriptome when assembling the transcriptome of Cadam-KW2161 using 13 different assembly methods. * indicates a known *C. adamanteus* transcript that was not detected in either Cadam-KW1942 or Cadam-KW2161, while ** indicates a known *C. adamanteus* transcript that was not detected among any of the five transcriptomes for this species analyzed in the current study.

Our accounting of the transcripts recovered by each assembler in each of two *C. adamanteus* transcriptomes further highlighted the striking amount of variation in the transcripts assembled. The strength of Extender in recovering SVMP and SVSP transcripts is most apparent in these analyses (Figure 5 and Figure 6), as no other approach recovered even half of the loci Extender was able to construct. Extender also consistently assembled several of the other loci that exist as single loci or small gene families, namely BPP, CRISP, LAAO, and PLA${}_{2}$, PLB, and Vespryn, respectively. However, Extender consistently failed to assemble 3FTx-1, 3FTx-2, HYAL-1, KUN-1, KUN-2, NUC-1, PDE-1, and VEGF-2, and missed MYO-2 and NGF-1 in one of the two assemblies, despite their clear presence in the transcriptome as made evident by recovery by several of the other assembly methods. This leaves open the prospect of choosing one to two complimentary transcriptome assemblers to augment Extender assemblies to achieve the most complete transcriptome possible.

Of these small gene family toxin transcripts not recovered by Extender, but present in other assemblies, Trinity recovers 100% of these in both *C. adamanteus* transcriptomes. Meanwhile, BinPacker fails to recover 3FTx-1 in Cadam-KW1942 and MYO-2 in Cadam-2161, and NGen14 fails to recover NGF-1, NUC-1 or MYO-2 in Cadam-KW1942 and PDE-1 in either of the samples. The only remaining contigs present in the global set of transcripts that would not have been recovered by a combined Extender and Trinity transcript set are CTL-7 and SVSP-8, which were both recovered by NGen14. Thus, a combined set of contigs from Extender, NGen14, and Trinity would provide the most complete set of *C. adamanteus* transcipts, although providing a higher number of seed reads to Extender could possibly lead to the recovery of CTL-7 and SVSP-8.

The various iterations of SPAdes were the least effective assembly method for *C. adamanteus*, recovering as few as two toxin transcripts in the case of SPAdes_k31. As in the quality assessment analyses above, more quality transcripts were recovered by SPAdes and SDT when higher *k*-mer values were used for assembly, pointing toward *k*-mer size as a key parameter when assembling multi-isoform data, as has been shown elsewhere [37,39].

### 2.4. Scorpion Assembly Quality

**Figure 7:**
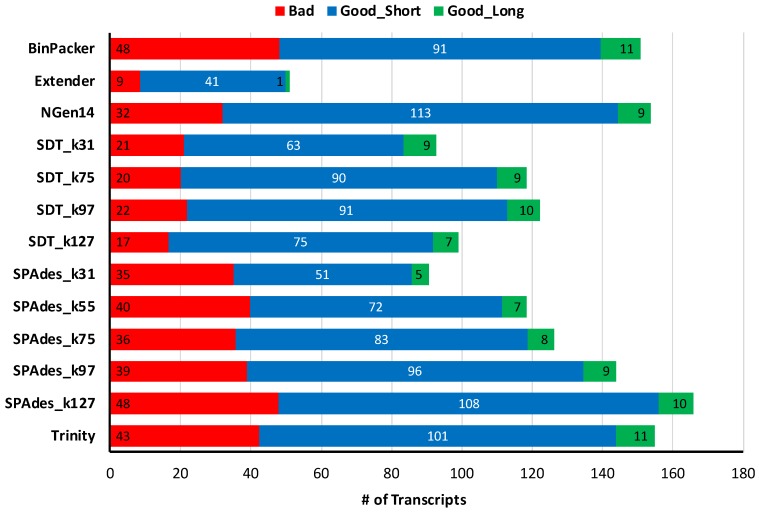
Average number of scorpion contigs classified as chimeric, Good_Short (<500 bp in length), or Good_Long (>500 bp in length), for each of 13 assembly methods.

Scorpions generally have much higher numbers of toxin loci than snakes, with distinct loci numbering in the hundreds [48]. We showed that similar numbers of high quality transcripts of greater than 500 bp in length are recovered by several assemblers (9–11 transcripts), including BinPacker, NGen14, SDT_k31, SDT_k75, SDT_k97, SPAdes_k97, SPAdes_k127, and Trinity (Figure 7). The recovery of quality transcripts below 500 bp in length varied extensively, with NGen14 recovering the most transcripts on average (113), followed by SPAdes_k127 (108), and Trinity (101). Extender recovered the lowest number of transcripts overall (mean = 51), and only an average of 41 of these passed quality filtering. In the poorer performance of Extender, the optimal assembly of scorpion toxin transcripts presents a marked deviation from that observed for snake transcriptomes, where Extender was most successful.

## 3. Discussion

Transcriptome studies of venomous animals provide the opportunity for the broad scale comparative analysis of evolutionary processes across the tree of life in a trait with a common purpose, that of harming other animals for predation or defense. However, the rise of next-generation sequencing for high-throughput acquisition of transcriptome data has been followed by an explosion in the available methods for assembling transcriptomes. Many studies to date have used only one assembly software to recover venom gene transcripts [27,30,31,32,49], raising the concern that biases in the relative performance of assembly software could lead to poor recovery of the complete set of transcripts and bias results on expression values toward those transcripts that are recovered by the researchers’ software of choice. Our results here confirm these fears, as we demonstrate strong differences in the performance of various assemblers that, importantly, are non-random with regard to the ability of each assembler to recover transcripts belonging to particular venom gene families. Variation in assembler performance can occur in (1) ability to recover the full suite of transcripts present in the face of expression variation and multi-isoform data, (2) biases in which toxin gene families are recovered, and (3) differences in the numbers of chimeric or otherwise misassembled transcripts that are produced. We find that combining the output of multiple assemblers and careful curation of the transcript set can circumvent these challenges.

One of the challenges of selecting an assembly method is the incongruence between many commonly used metrics of transcriptome assembly quality and performance in the assembly of toxin genes. Simple metrics such as various derivatives of contig length and read mapping proportions are commonly cited during assembly choice [21,27,30,32,35], and methods such as BUSCO and Transrate have emerged to harness these and other measures for powerful evaluations of whole assembly quality and completeness [47,50]. However, our findings demonstrate that those assemblers which perform best by these traditional classifications do not necessarily assemble toxins effectively (or at all in the case of some gene families). The SDT and SPAdes assemblers both recovered substantial numbers of BUSCO orthologous nontoxin loci and had high transrate scores, but assembled few toxin loci, a substantial proportion of which were chimeric misassemblies. Conversely, the seed-and-extend approach of Extender was only effective for toxin gene recovery as implemented here, resulting in few BUSCO nontoxin loci recovered and low Transrate scores. The low Transrate scores resulted from an overall low proportion of total reads mapping, since only toxin genes were assembled. Clearly, evaluating the quality of toxin gene assemblies requires a toxin-focused approach, and thus some prior knowledge of what to expect in the transcriptome of the focal taxa. Running programs like Transrate on only the putative toxin genes, as we do as a chimera filtering step here, can supplement manual inspection of coverage profiles as a way to evaluate individual contigs for signs of misassembly.

The impacts of *k*-mer size on the recovery of quality transcripts during de novo assembly have been previously explored, although not specifically for toxin genes. In previous studies, longer *k*-mer sizes lead to fewer transcripts overall due to missing low abundance transcripts [13,51], although the transcripts that are recovered are less likely to be misassembled [51,52]. In our venom gland transcriptomes, larger *k*-mer sizes lead to the recovery of fewer complete and single-copy BUSCO loci, supporting the trends seen in previous work. However, more toxin transcripts, overall, tended to be recovered at higher *k*-mer sizes. Since even rare toxins are often highly expressed compared to non-toxin loci, the gains in using small *k*-mer sizes for assembling rare transcripts may not be as applicable to toxin loci in venom-gland tissue. Despite this, there were toxin contigs that were sometimes recovered when using small *k*-mer sizes that were not recovered when using a longer *k*-mer. Our completeness analyses of two *C. adamanteus* transcriptomes are informative in this regard, as the largest *k*-mer size for both SDT and SPAdes assemblies provided the highest numbers of total toxins, but there was some complementarity provided by the analyses done at smaller *k*-mer sizes as well (between four and eight toxins detected with only smaller values of *k* in SDT or SPAdes assemblies). Thus, it is still important to consider whether assemblies using different *k*-mer parameters should be combined (i.e., combining results of SDT or SPAdes using varying *k*-mer size parameters) in a final assembly. Combining *k*-mer sizes during assembly has been advocated previously [13,51] and appears to be advisable for the recovery of more complete sets of toxin loci as well if the assembly software permits varying this parameter. However, our results agree most broadly with those of Rana et al. [39] in showing that the major driver of transcript recovery is the choice of assembly software and less so the choice of *k*-mer length.

Our assessments of assembly completeness in two individuals *C. adamanteus* showed that gaps in assembler performance were non-random, and, instead, that different assemblers excelled in the recovery of particular toxin classes. This effect was most prominent for CTLs, SVMPs, and SVSPs. For these toxin gene familes, most assemblers recovered one or two unique transcripts for each toxin class, but Extender recovered several isoforms. The most likely cause of these performance differentials is that these are the three largest gene families in the pitvipers analyzed herein, with ten or more isoforms of each family appearing in some individuals (e.g., Figure 5). The seed-and-extend approach of Extender accurately recovered these transcripts by requiring large areas of perfect overlap during extension of a contig. Another assembly program, VTBuilder, implements its own seed, cluster, and extension algorithm and has been reported to recover multi-isoform venom toxin transcripts such as SVMPs well [53]. Future work comparing the extent to which Extender and VTBuilder overlap or complement one another would be interesting, although VTBuilder is currently limited to datasets of five million reads or less. Meanwhile, assemblers like Trinity, which are known to perform poorly with multi-isoform data by collapsing several isoforms into a single output contig [40], were likely confounded by several equally likely possible paths in their assembly algorithm, producing chimeras or nothing at all. The tendency of assemblers to recover one or two isoforms of these key toxin classes, some of which may have >20 copies [54] in the genome, is disconcerting and would likely lead to vastly misleading conclusions regarding transcriptome composition. Conversely, the de Brujn graph methods, along with BinPacker and NGen14, showed improved performance for those toxins that exist in single or low-copy toxin families. BinPacker, NGen14, and Trinity showed particular efficiency in the recovery of those small family toxins when the toxins were also low in their relative expression in the transcriptome. Meanwhile, Extender failed to assemble several of these small-family, low-expression toxins in *C. adamanteus* and produced fewer of them overall across both snakes and scorpions. We found high complementarity in the way the chosen assemblers performed, with Extender recovering those toxins that occurred in large gene families and a smattering of the remaining toxins, with the remaining transcripts assembled by other assemblers such as Trinity and NGen14.

The clear message of these findings is that no single assembler recovered all toxin loci present, and thus accurate venom gland transcriptome studies should combine the quality-filtered output of multiple assembly approaches to obtain a complete set of toxin transcripts. Recovering a high-quality transcriptome assembly through clustering and merging assemblies recovered through different methods has been shown to be effective in other systems [55], and our results suggest this strategy would provide an effective solution here. There are a variety of methods for clustering transcripts based on sequence identity and/or inferred homology [56], including pipelines that implement internal quality evaluation methods like Transrate [57]. Although developing such a bioinformatic pipeline for venom gland transcriptomes may seem advantageous, the high variability in transcript quality and recovery we observed across taxa and the apparent unreliability of many quality metrics suggest that such an undertaking is premature.

For instance, among the approaches used here, Extender was crucial to the complete assembly of pitviper transcriptomes, but it produced by far the fewest total high quality toxins for the scorpion assemblies. The scorpions, *C. hentzi* and *H. spadix*, are known to express hundreds of toxin loci in their venom glands [48,58]. This diversity likely explains the apparent poor performance of the seed-and-extend approach of Extender for these scorpions, as we provided only 1000 seed reads (as we did for snakes). If a transcript is represented by only 1000 reads in our roughly 10 million read samples, the chance of sampling this transcript is 1 in 10 for assembly via extension. Hence, the complexity of a venom-gland transcriptome merits consideration with this approach, where expectations for the appropriate number of seeds to sample all of the transcripts should be calculated. With this degree of variability among single assembly performance, which one would not necessarily expect a priori, we suggest the responsibility of selecting appropriate assembly and clustering strategies should remain with the researcher as opposed to a “one-size-fits-all” pipeline. This is particularly true when investigating novel or understudied taxonomic groups where a pipeline’s implicit assumptions about the structure and content of the toxin repertoire could lead to the recovery of a mediocre assembly, but inaccurate perception of its quality. However, the maintenance of other trends, such as the high performance of BinPacker, NGen14, and Trinity and improvements at higher *k*-mer values, suggest that these factors impacting assembler choice can be safely transferred among diverse venomous taxa.

The emergence of cheaper genomes promises more frequent use of referenced-based transcriptome assembly in the future, even for non-model organisms. The complex and dynamic genetic architecture of toxin genes, however, will most likely require de novo assemblies, maintaining the de novo approach as a key tool in the study of animal venoms for some time to come. We have demonstrated the possibility for serious misrepresentation of the composition and diversity of venom-gland transcriptomes if care is not taken during assembly; even careful work using a single assembly method can recover mere fractions of the important transcripts involved in toxin production. Our practical recommendations for the venom evolutionary biologist or toxinologist are to combine assemblers that employ wholly different assembly strategies and to pool their output instead of choosing a “best” assembler, particularly if basing the choice on any traditional metric of assembly quality that is not directly related to toxin transcript recovery. Instead, iterative evaluation of your assemblies with available data from genomes, previously published transcriptomes, and proteomes can help ensure high quality assemblies of venom gland transcriptomes for accurate studies of animal venom systems.

## 4. Materials and Methods

### 4.1. Sample Collection and Transcriptome Sequencing

We utilized venom gland RNA-seq reads from eleven snakes and four scorpions (Table 1). All reads were 150-bp paired-end reads generated on a HiSeq 2500 in the Translational Science Laboratory in the College of Medicine at Florida State University. The reads for the five individuals of *C. adamanteus* were generated by Rokyta et al. [43]. The reads for *H. spadix* [58] and *C. hentzi* [48] were also derived from previous work. The remaining snake data sets (one *A. piscivorus*, five *Crotalus adamanteus*, one *C. cerastes*, one juvenile *C. horridus*, one adult *C. horridus*, one *Sistrurus catenatus*, and one *S. miliarius*) represented new RNA-seq libraries and reads generated from previously published gland tissues [59].

### 4.2. Tissue Preparation and Sequencing

Venom glands of both snakes and scorpions used in this study were extracted using previously described protocols [48,60]. All glands were extracted four days after venom extraction (milking) to stimulate transcription of venom genes [61]. Venom gland RNA extraction was accomplished with Trizol (Invitrogen, Carlsbad, CA, USA) extraction, where homogenized gland tissue was transferred to 500 μL of Trizol. The tissue and Trizol were mixed by aspiration with a 20 gauge needle, followed by the addition of 500 μL additional Trizol and 200 μL of chloroform. The mixture was transferred to phase lock heavy gel tubes (5Prime) to separate the RNA and additional cellular components. The addition of isopropyl alcohol was used to precipitate the RNA, which was then washed with 75% ethanol and dissolved in Ultrapure water (Invitrogen). The concentration of the RNA was determined with the Qubit Broad Range RNA assay (Thermo-Fisher Scientific, Waltham, MA, USA) and quality of the extraction was checked on a Bioanalyzer using the RNA 6000 Pico Kit (Agilent Technologies, Santa Clara, CA, USA).

Total RNA from venom glands, with equimolar pooling of right and left venom gland RNA from the snakes, was used for mRNA isolation with the NEBNext Poly(A) mRNA Magnetic Isolation Module (New England Biolabs, Ipswich, MA, USA), followed by the preparation of cDNA libraries using the NEBNext Ultra RNA Library Prep Kit (New England Biolabs) and Multiplex Oligos for Illumina (New England Biolabs), according to the manufacturer’s instructions. Purification of resultant cDNA was carried out with Agencourt Ampure XP PCR Purification Beads (Beckman Coulter, Inc., Brea, CA, USA). Library quality was assessed using a Bioanalyzer with the High Sensitivity DNA Kit (Agilent Technologies). KAPA PCR was performed to assess the amplifiable concentration of cDNA libraries, followed by the sequencing of 5 nM per sample pooled libraries on an Illumina HiSeq 2500 at Florida State University with 150 bp paired-end reads.

### 4.3. Transcriptome Assemblies

Raw reads were checked for potential sample cross-leakage through index misassignment within sequencing lanes. Counts of all 57-mers in the raw reads for each sample in each lane were generated with Jellyfish v. 2.2.6 [62], and 57-mers that showed >500× count differentials between each pair of samples in a lane were identified. Reads with 25% or more of their lengths comprised of 57-mers in this set were removed from the sample with lower counts. We used Trim Galore! v. 0.4.4 [63] for adapter and quality trimming with a quality threshold of phred 5. Trimmed reads less than 75 nucleotides were removed. Overlapping reads were merged with PEAR v. 0.9.10 [64].

To compare assembly methods, we chose several assemblers commonly used for de novo transcriptome assemblies, and assembled the same short-read RNA-seq data with each assembler. Three of these assemblers used variations of the de Bruijn graph approach to contig construction: rnaSPAdes (SPAdes) [15], SOAPdenovo-Trans (SDT) [12], and Trinity [46]. We also employed BinPacker [45], an assembler that incorporates coverage information to construct its splicing graphs and is reported to perform well with multi-isoform data. Additionally, we employed SeqMan NGen v. 14 (DNAStar, Inc., Madison, WI, USA; NGen14), which is also a non-de Bruijn graph method based on proprietary algorithms. Finally, we used an in-house assembler, Extender [33], which randomly selects seed reads and extends these outward based on matching overlap with other reads to form contigs. The VTBuilder [53] assembler implements a similar seed-and-extension algorithm in its approach to multi-isoform transcript assembly. However, its current limit of five million input reads renders it unsuitable for the current scale of RNA-seq datasets (21–48 million reads per sample for our data), and we therefore did not evaluate it. Assembly methods differed in the format of input reads and therefore in overall read counts used for each assembly. BinPacker, SPAdes, SDT, NGen14, and Trinity used both merged and unmerged read pairs, whereas Extender used only the merged read pairs. BinPacker, NGen14, and Trinity treated all reads as unpaired, whereas SDT and SPAdes treated unmerged reads as paired reads.

We ran SDT v. 1.03 at four *k*-mer sizes: *k* = 31, *k* = 75, *k* = 95, and *k* = 127 with each run saved as an independent assembly. Maximum and minimum read lengths were set at 500 and 200 bp, respectively, with an average insert size of 250 bp. Similarly, we generated assemblies with rnaSPAdes in SPAdes v. 3.9.0 using the same four *k*-mer sizes used in SDT assemblies with the addition of an assembler at *k*-mer size of *k* = 55, the default *k*-mer size for SPAdes. We used SPAdes default settings for assembly. For trinity assemblies, we ran Trinity v. 2.4.0 with a minimum contig length of 200 bp and a *k*-mer size of *k* = 31. BinPacker v. 1.0 was similarly run with a *k*-mer size of *k* = 31, as Liu et al. [45] suggested that larger *k*-mer sizes than the default *k* = 25 might produce better assemblies when using longer reads. We ran NGen14 with default settings. Finally, we ran our only seed and assemble approach, Extender [33], with 1000 randomly selected seeds with a minimum quality of 30 at all base positions. Seeds were prohibited from sharing any *k*-mers as long as the extension-overlap length (120 bp). We required at least two extensions for each direction to retain the results of the seed. We set a 120 bp minimum overlap for extension and a minimum quality score of at least 20 at all base positions for a read to be considered for extension. We allowed 20 replicates per seed per direction and required that 20% of replicates per seed be extended in order retain a seed.

### 4.4. Full Assembly Quality and Recovery of Nontoxins

To evaluate each assembly with traditional metrics, and because nontoxin genes can be valuable for establishing baselines for studies of evolutionary rates and phylogenetics, we compared each assembly using two common metrics of assembly quality. We used the program BUSCO v. 3 to identify single-copy, orthologous nontoxin loci in each assembly [44,50]. BUSCO compares assembled contigs against lineage specific subsets of the OrthoDB v.9 [65] database using tBLASTn [66], followed by HMMER [67] classification of annotated contigs as complete and single copy, complete and duplicated, fragmented, or missing. OrthoDB ortholog sets contain genes that exist as a single-copy in the genome of 90% of the species in the database, and thus provides an evolutionary expectation of presence in an assembled gene set if the assembly is complete. For a transcriptome study, all loci are not expected to be present due to lack of expression in the target tissue, but a BUSCO analysis will permit quantitative comparison of multiple assemblies of the same transcriptome in terms of the overall number of complete and single-copy orthologous loci recovered out of the full set of loci in the OrthoDB reference database. For BUSCO analysis of the snake venom gland transcriptome assemblies, we used the Tetrapoda ortholog set containing 3950 loci, whereas we analyzed our scorpion assemblies using the Arthropoda ortholog set of 1066 loci. Our criteria for ranking nontoxin assembly quality is simply determining which assembler yielded the highest number of complete and single-copy matches to the OrthoDB loci.

For a second assessment of assembly quality, we ran Transrate v. 1.0.3 [47]. Transrate assigns a score to each assembled contig based on read mapping statistics that allow the base calls and structure of the contig to be evaluated for chimerism or other forms of misassembly. The final “Transrate score” is an assembly quality score (from 0–1, with 1 being highest quality) that is an average measure of all contig quality scores weighted by the proportion of reads that map to the assembled contigs.

### 4.5. Evaluating Toxin Gene Assembly

We evaluated the quality of toxin transcript assemblies based on the quality of toxin contigs and the completeness of the final transcript sets. To determine the number of high quality toxin transcripts assembled by each piece of assembly software, we employed a series of filtering steps to identify contigs that were (1) identified as toxin genes and (2) lacked signs of chimerism or fragmentation. First, we filtered the assemblies to remove contigs less than 150 bp in length, as venom genes shorter than this have not been identified in snakes or scorpions. Next, we matched the assembled contigs to internally curated databases of snake or scorpion toxin coding sequences using the clustering program cd-hit-est [56] at a global match percentage of 80%. Any transcript meeting this similarity threshold and matching one of the toxin coding sequences in the reference database end-to-end was annotated as such. The coding regions of the annnotated toxin contigs were then clustered using cd-hit-est at a match percentage of 98% to remove duplicate sequences and allelic variation, following Ward et al. [48].

To derive a final set of unique and high quality toxin sequences, we applied three filtering criteria to the coding regions of our annotated, clustered contigs. First, we aligned the merged, paired-end reads to the coding regions using BWA v. 0.7.16 [68], allowing reads to map only if zero mismatches occurred in the alignment. These alignments were used to obtain coverage information across the entire contig using bedtools2 [69] and to calculate the probability that the contig represents a single (i.e., non-chimeric) transcript using the program Transrate v. 1.0.3 [47]. We retained only those contigs that (1) had coverage >0 across all bases in the coding region, (2) had <100-fold coverage differentials across the length of the coding sequence, and (3) had a Transrate “segmentation probability” of non-chimerism >0.9. We then counted the number of chimeric and high-quality toxin genes produced by each assembler for each toxin gene family. For the scorpion data, we classified toxin sequences as short or long based a cutoff size of 1000 bp to explore differences in the ability of each assembler to assemble transcripts of varying length. For snakes, we classified toxins according to gene family members to explore the ability of each assembler to assemble transcripts of each venom gene family when present in the transcriptome.

Finally, we used two of our *C. adamanteus* individuals to gain measures of assembly completeness and complementarity of assemblers in recovering the full set of transcripts identified to date in *C. adamanteus* [43]. For this analysis, we used the set of toxin coding sequences of Cadam-KW1942 and Cadam-KW2161 after clustering at 98%. We did not apply our quality filtering steps to these individuals as described above; this allowed us to identify toxin presence/absence in more detail. The *C. adamanteus* toxin contigs were then annotated on the basis of 98% sequence similarity to a comprehensive set of 59 consensus *C. adamanteus* toxin coding sequences pooled from several previously published transcriptomes, including those analyzed herein [43], again using cd-hit-est for clustering contigs and the reference database. Contigs with a match ≥98% in the reference transcript set were marked as present in the focal assembly, and this analysis was repeated for the output of each assembly method in both *C. adamanteus* individuals.

## Figures and Tables

**Table 1:** Samples and raw Illumina data.

Species	Sample ID	Read Pairs	Short Read Archive Accession
**Pitvipers**
*Agkistrodon piscivorus*	KW1750	17,165,966	SRR6916474
*Crotalus adamanteus* #1	KW1942	23,568,901	SRR5259496, SRR5259495
*Crotalus adamanteus* #2	KW2161	19,500,323	SRR5259494, SRR5259493
*Crotalus adamanteus* #3	KW2170	10,758,708	SRR5259492, SRR5259491
*Crotalus adamanteus* #4	KW2171	15,862,828	SRR5259490, SRR5259489
*Crotalus adamanteus* #5	MM0127	13,497,975	SRR5259486, SRR5259485
*Crotalus cerastes*	KW1744	17,607,533	SRR6768689
*Crotalus horridus*-Juvenile	KW1369	12,409,168	SRR6916473
*Crotalus horridus*-Adult	KW1089	14,798,031	SRR6916472
*Sistrurus catenatus*	KW1090	13,171,023	SRR6916476
*Sistrurus miliarius*	KW1749	15,292,491	SRR6916477
**Scorpions**
*Centruroides hentzi* #1	C0136	18,977,620	SRR6041834
*Centruroides hentzi* #2	C0148	20,032,620	SRR6041835
*Hadrurus spadix* #1	C0195	12,205,269	SRR4069277
*Hadrurus spadix* #2	C0196	24,243,211	SRR4069278

**Table 2:** Counts of transcripts assembled for eight species of pitviper snake. *Crotalus adamanteus* counts are mean (range) derived from four individuals. A dash (—) denotes a toxin class that was not assembled. Several functionally important toxin classes, including C-type lectins (CTL), phospholipases (PLA2), snake venom serine proteases (SVSP), and snake venom metalloproteinases (SVMP), are tabulated separately.

Species/Assembly	CTL	PLA2	SVSP	SVMP	Other Toxins	Total Toxins
***Agkistrodon contortrix***						
BinPacker	5	2	—	—	16	23
Extender	8	3	11	11	9	42
NGen14	9	5	8	6	12	40
SDT_k31	—	2	—	—	12	14
SDT_k75	3	4	1	—	12	20
SDT_k97	5	4	2	—	13	24
SDT_k127	5	3	5	1	8	22
SPAdes_k31	1	1	—	—	7	9
SPAdes_k55	1	1	—	1	8	11
SPAdes_k75	5	1	—	2	8	16
SPAdes_k97	4	1	5	5	12	27
SPAdes_k127	5	1	6	3	12	27
Trinity	7	3	—	1	17	28
***Crotalus adamanteus***						
BinPacker	2.6 (1–5)	1.8 (1–2)	1.0 (1–1)	0.2 (0–1)	16.6 (14–19)	21.6 (19–25)
Extender	8.4 (6–11)	1.4 (1–2)	7.2 (2–11)	5.0 (2–7)	5.6 (2–9)	27.6 (13–34)
NGen14	9.2 (5–12)	1.8 (1–2)	3.4 (1–5)	0.4 (0–2)	12.4 (8–16)	27.2 (22–33)
SDT_k31	1.0 (1–1)	1.0 (1–1)	—	—	10.0 (8–13)	11.4 (10–14)
SDT_k75	4.4 (2–6)	1.2 (1–2)	1.2 (1–2)	—	11.4 (10–15)	18.8 (15–22)
SDT_k97	6.8 (5–8)	2.0 (1–2)	2.0 (1–3)	—	11.8 (9–15)	22.2 (17–27)
SDT_k127	6.4 (6–7)	1.3 (1–2)	3.3 (2–5)	0.2 (0–1)	10.4 (8–14)	20.6 (16–24)
SPAdes_k31	1.0 (1–1)	—	—	—	3.6 (2–8)	4.0 (2–9)
SPAdes_k55	2.3 (1–3)	2.0 (2–2)	—	—	5.0 (3–7)	7.2 (4–12)
SPAdes_k75	2.7 (2–3)	1.0 (1–1)	2.3 (1–4)	—	7.0 (5–11)	10.2 (5–16)
SPAdes_k97	2.6 (1–6)	1.0 (1–1)	2.7 (1–4)	—	7.4 (5–12)	11.8 (6–21)
SPAdes_k127	3.0 (1–6)	1.0 (1–1)	2.8 (1–5)	0.8 (0–2)	12.2 (7–23)	18.6 (8–33)
Trinity	6.8 (3–11)	1.8 (1–2)	2.0 (2–2)	—	17.4 (12–21)	26.0 (21–34)
***Crotalus cerastes***						
BinPacker	5	1	—	—	16	23
Extender	6	1	9	10	5	32
NGen14	12	1	7	1	13	35
SDT_k31	3	1	—	—	11	16
SDT_k75	10	1	1	—	13	26
SDT_k97	11	1	4	—	11	27
SDT_k127	12	—	4	1	8	26
SPAdes_k31	4	—	—	—	7	12
SPAdes_k55	4	—	2	—	8	14
SPAdes_k75	11	—	4	—	6	22
SPAdes_k97	7	—	3	1	8	20
SPAdes_k127	10	—	6	1	13	31
Trinity	6	1	1	—	16	26
***Crotalus horridus***—Juvenile						
BinPacker	4	1	—	—	15	21
Extender	4	1	9	6	4	24
NGen14	6	—	6	1	11	25
SDT_k31	1	1	—	—	11	13
SDT_k75	2	1	2	—	10	17
SDT_k97	4	1	3	1	10	21
SDT_k127	5	1	5	1	6	18
SPAdes_k31	2	—	—	—	5	7
SPAdes_k55	2	—	—	—	7	10
SPAdes_k75	5	—	2	—	9	17
SPAdes_k97	6	—	3	—	9	19
SPAdes_k127	6	—	1	3	8	19
Trinity	7	1	—	—	13	22
***Agkistrodon contortrix***						
BinPacker	5	2	—	—	16	23
Extender	8	3	11	11	9	42
NGen14	9	5	8	6	12	40
SDT_k31	—	2	—	—	12	14
SDT_k75	3	4	1	—	12	20
SDT_k97	5	4	2	—	13	24
SDT_k127	5	3	5	1	8	22
SPAdes_k31	1	1	—	—	7	9
SPAdes_k55	1	1	—	1	8	11
SPAdes_k75	5	1	—	2	8	16
SPAdes_k97	4	1	5	5	12	27
SPAdes_k127	5	1	6	3	12	27
Trinity	7	3	—	1	17	28
***Crotalus adamanteus***						
BinPacker	2.6 (1–5)	1.8 (1–2)	1.0 (1–1)	0.2 (0–1)	16.6 (14–19)	21.6 (19–25)
Extender	8.4 (6–11)	1.4 (1–2)	7.2 (2–11)	5.0 (2–7)	5.6 (2–9)	27.6 (13–34)
NGen14	9.2 (5–12)	1.8 (1–2)	3.4 (1–5)	0.4 (0–2)	12.4 (8–16)	27.2 (22–33)
SDT_k31	1.0 (1–1)	1.0 (1–1)	—	—	10.0 (8–13)	11.4 (10–14)
SDT_k75	4.4 (2–6)	1.2 (1–2)	1.2 (1–2)	—	11.4 (10–15)	18.8 (15–22)
SDT_k97	6.8 (5–8)	2.0 (1–2)	2.0 (1–3)	—	11.8 (9–15)	22.2 (17–27)
SDT_k127	6.4 (6–7)	1.3 (1–2)	3.3 (2–5)	0.2 (0–1)	10.4 (8–14)	20.6 (16–24)
SPAdes_k31	1.0 (1–1)	—	—	—	3.6 (2–8)	4.0 (2–9)
SPAdes_k55	2.3 (1–3)	2.0 (2–2)	—	—	5.0 (3–7)	7.2 (4–12)
SPAdes_k75	2.7 (2–3)	1.0 (1–1)	2.3 (1–4)	—	7.0 (5–11)	10.2 (5–16)
SPAdes_k97	2.6 (1–6)	1.0 (1–1)	2.7 (1–4)	—	7.4 (5–12)	11.8 (6–21)
SPAdes_k127	3.0 (1–6)	1.0 (1–1)	2.8 (1–5)	0.8 (0–2)	12.2 (7–23)	18.6 (8–33)
Trinity	6.8 (3–11)	1.8 (1–2)	2.0 (2–2)	—	17.4 (12–21)	26.0 (21–34)
***Crotalus cerastes***						
BinPacker	5	1	—	—	16	23
Extender	6	1	9	10	5	32
NGen14	12	1	7	1	13	35
SDT_k31	3	1	—	—	11	16
SDT_k75	10	1	1	—	13	26
SDT_k97	11	1	4	—	11	27
SDT_k127	12	—	4	1	8	26
SPAdes_k31	4	—	—	—	7	12
SPAdes_k55	4	—	2	—	8	14
SPAdes_k75	11	—	4	—	6	22
SPAdes_k97	7	—	3	1	8	20
SPAdes_k127	10	—	6	1	13	31
Trinity	6	1	1	—	16	26
***Crotalus horridus***—Juvenile						
BinPacker	4	1	—	—	15	21
Extender	4	1	9	6	4	24
NGen14	6	—	6	1	11	25
SDT_k31	1	1	—	—	11	13
SDT_k75	2	1	2	—	10	17
SDT_k97	4	1	3	1	10	21
SDT_k127	5	1	5	1	6	18
SPAdes_k31	2	—	—	—	5	7
SPAdes_k55	2	—	—	—	7	10
SPAdes_k75	5	—	2	—	9	17
SPAdes_k97	6	—	3	—	9	19
SPAdes_k127	6	—	1	3	8	19
Trinity	7	1	—	—	13	22

## Abbreviations

The following abbreviations are used in this manuscript:

3FTx	three-finger toxin
bp	base pair
BPP	bradykinin potentiating peptide
CRISP	cysteine rich secretory protein
CTL	C-type lectin
HYAL	hyaluronidase
KUN	Kunitz-like protein
LAAO	L-amino acid oxidase
NGen14	SeqMan NGen v.14
MYO	myotoxin
PDE	phosphodiesterase
PLA2	phospholipase A2
PLB	phospholipase B
SDT	SOAPdenovo-Trans
SPAdes	rnaSPAdes
SVMP	snake venom metalloproteinase
SVSP	snake venom serine protease
VESP	vespryn
VEGF	vascular endothelial growth factor
